# Clinical potential of hemodynamic ramp test by simultaneous echocardiography and right heart catheterization for aortic insufficiency in a patient with continuous-flow left ventricular assist device

**DOI:** 10.1007/s10047-020-01210-y

**Published:** 2020-09-17

**Authors:** Yasumori Sujino, Kensuke Kuroda, Koichi Yoshitake, Nobuichiro Yagi, Eiji Anegawa, Hiroki Mochizuki, Keiichiro Iwasaki, Seiko Nakajima, Takuya Watanabe, Masanobu Yanase, Satsuki Fukushima, Tomoyuki Fujita, Junjiro Kobayashi, Norihide Fukushima

**Affiliations:** 1 Department of Transplant Medicine, National Cerebral and Cardiovascular Center, 6-1 Kishibe-shinmachi, Suita, Osaka 564-8564 Japan; 2 Department of Cardiovascular Surgery, National Cerebral and Cardiovascular Center, 6-1 Kishibe-Shinmachi, Suita, 564-8564 Osaka Japan

**Keywords:** Heart-assist devices, Aortic valve replacement, Aortic valve insufficiency, Cardiac output, Pulmonary wedge pressure

## Abstract

Aortic insufficiency (AI) is an important adverse event in patients with continuous-flow (CF) left ventricular assist device (LVAD) support. AI is often progressive, resulting in elevated 2-year morbidity and mortality. The effectiveness of echocardiographic ramp studies in patients with AI has been unclear. Here, we describe a patient with a CF-LVAD implant who underwent aortic valve replacement (AVR), following assessment of AI using a hemodynamic ramp test with simultaneous echocardiography and right heart catheterization (RHC). The patient was a 21-year-old man with cardiogenic shock due to acute myocarditis, who underwent HeartWare CF-LVAD (HVAD) implantation. Heart failure persisted despite increased doses of diuretics and inotrope, as well as an increased HVAD pump rate. HVAD monitoring revealed a correlation between increased HVAD pump rate and flow at each speed step. A hemodynamic ramp test with simultaneous transthoracic echocardiography and RHC revealed a significant discrepancy between HVAD pump flow and cardiac output (CO) at each speed step; moreover, pulmonary capillary wedge pressure remained high. Therefore, the patient underwent AVR. Subsequently, his low CO symptoms disappeared and inotropes were successfully discontinued. A postoperative hemodynamic ramp test revealed that AVR had successfully closed the loop of blood flow and reduced the discrepancy between HVAD pump flow and CO, thereby increasing CO. The patient was then discharged uneventfully. In conclusion, a hemodynamic ramp test with simultaneous echocardiography and RHC was useful for the evaluation of the causal relationship between AI and low CO, and for selection of surgical treatment for AI in a patient with CF-LVAD.

## Introduction

Aortic insufficiency (AI) is an important adverse event in patients with continuous-flow (CF) left ventricular assist device (LVAD) support [1]. In such patients, survival is considerably worse when de novo AI develops during long-term LVAD [2]. This is presumed to be at least partially due to changes in shear stress across the aortic valve after CF-LVAD implantation [3, 4]. In addition, AI is often progressive, resulting in blood return to the left ventricle via a low-resistance circuit that leads to insufficient LVAD support, end-organ malperfusion, and multiple organ dysfunction syndrome in patients with heart failure [4]. The development of AI in this population is associated with elevated 2-year morbidity and mortality [2]. However, surgical indications for AI remain controversial. To evaluate the effect of AI on LVAD support, echocardiographic ramp studies may be useful; however, the effectiveness of these studies in patients with AI has not been elucidated [5]. Here, we describe a patient with a CF-LVAD implant who underwent aortic valve replacement (AVR), following assessment of the degree of AI using a hemodynamic ramp test with simultaneous echocardiography and right heart catheterization (RHC).

## Case report

A 21-year-old man with cardiogenic shock due to acute myocarditis was urgently transferred to our hospital with intra-aortic balloon pump and veno-arterial extracorporeal membrane oxygenation support. On admission, the patient’s temperature was 37.9 ℃. His blood pressure was 90/53 mmHg with a regular pulse rate of 78 beats/min, under intra-aortic balloon pump and veno-arterial extracorporeal membrane oxygenation support. Echocardiography demonstrated a left ventricular ejection fraction of 10%, mild aortic regurgitation, the tendency of noncoronary cusp’s prolapse, and severely reduced contraction of the right ventricle. Notable laboratory values on admission included creatinine kinase 2787 IU/L, creatinine kinase-MB 63.6 IU/L, aspartate aminotransferase 311 IU/L, alanine aminotransferase 54 IU/L, and creatinine 1.51 mg/dL. The patient immediately underwent central extracorporeal membrane oxygenation and closure of a patent foramen ovale. On his 7th day of hospitalization, the patient underwent biventricular assist device implantation using RotaFlow (Maquet Cardiovascular, Wayne, NJ, USA) in the left side and Biofloat (Nipro Corporation, Osaka, Japan) in the right side, as well as tricuspid valvuloplasty; subsequently, he recovered from profound cardiogenic shock. On the patient’s 41st day of hospitalization, the extracorporeal LVAD was modified to the HeartWare CF-LVAD (HVAD) (HeartWare International, Framingham, MA, USA) after he was identified as a heart transplant candidate. The RVAD was removed; however, heart failure persisted despite increased doses of diuretics and inotrope, as well as an increased HVAD pump rate (from 2500 to 3200 rotations per minute [rpm]). Echocardiography revealed both reduced ventricular contraction and moderate AI; blood examination revealed serum total bilirubin 1.6 mg/dL and brain natriuretic peptide 662.4 pg/mL.

To determine whether AI contributed to refractory low cardiac output syndrome despite CF-LVAD support, a hemodynamic ramp test with simultaneous echocardiography and RHC was performed. Briefly, the patient’s device speed was lowered to 2300 rpm. After 2 min, transthoracic echocardiographic images were obtained and hemodynamic parameters were measured by RHC; the following parameters were recorded: left ventricular end-diastolic dimension (LVEDD), pulmonary capillary wedge pressure (PCWP), and cardiac output (CO). In addition, the following pump parameters were recorded: power, pulsatility index, and pump flow calculated by HVAD monitoring. The device speed was then increased by 200 rpm at 2-min intervals with repeated acquisition of all echocardiographic, RHC, and device parameters at each speed step. The device speed was increased incrementally from 2300 to 3400 rpm. There were no suction events during the hemodynamic ramp test.

**Fig. 1:**
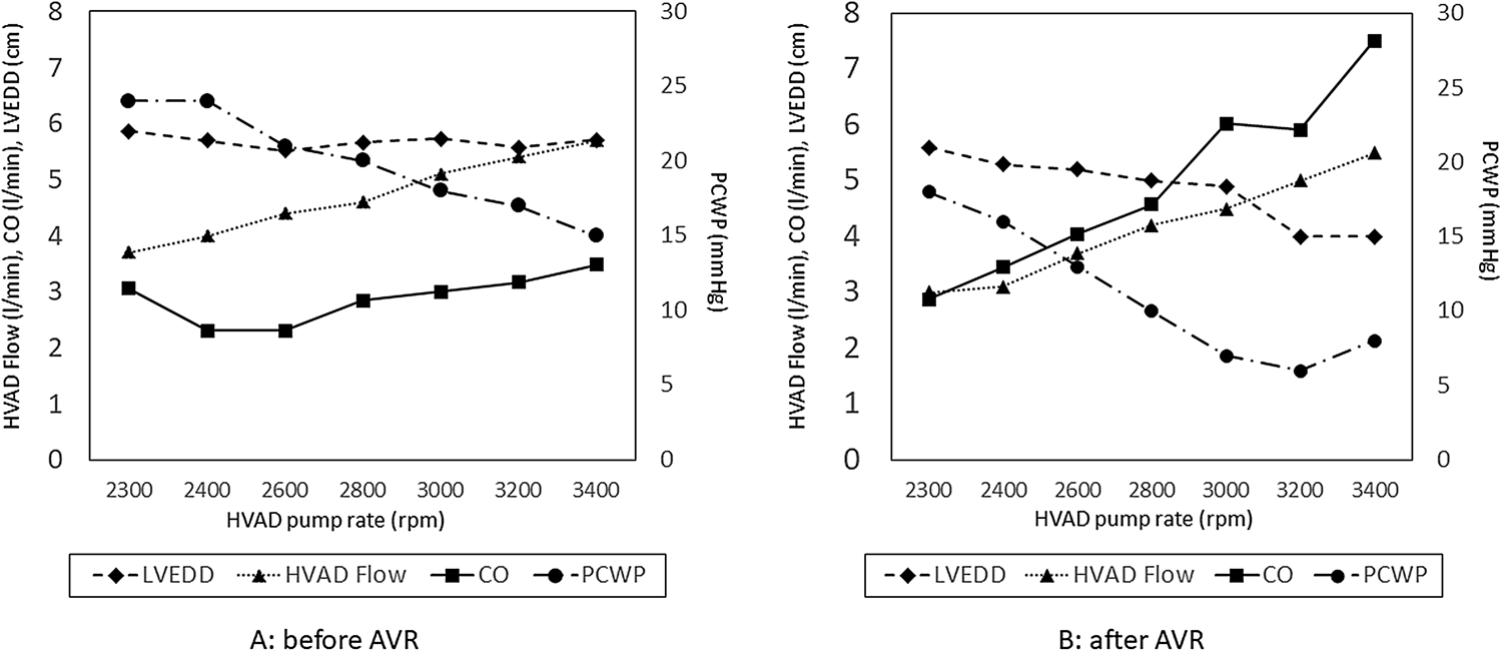
Results of hemodynamic ramp test before and after aortic valve replacement (AVR) (**a** and **b**). *LVEDD* left ventricular end-diastolic diameter, *HVAD flow* HeartWare continuous-flow left ventricular assist device pump flow calculated by ventricular assist device monitoring, *CO* cardiac output calculated by right heart catheterization, *PCWP* pulmonary capillary wedge pressure, *rpm* rotations per minute

Notably, the hemodynamic ramp test revealed only slight reduction in LVEDD for each speed increase of − 0.00081 cm/increment [6], as well as a discrepancy between HVAD pump flow and CO (3.05 vs 3.7 L/min at 2,400 rpm and 3.48 vs 5.7 L/min at 3400 rpm) (Table 1a and Fig. 1a). We presumed that this discrepancy between HVAD pump flow and CO was caused by blood returning to the left ventricle through the insufficient aortic valve. The patient underwent AVR on his 115th day of hospitalization. His symptoms (e.g., shortness of breath, dyspnea on exertion, and pretibial edema) rapidly disappeared. On the patient’s 122nd day of hospitalization, he was successfully weaned off intravenous inotropes. A hemodynamic ramp test conducted after AVR confirmed greater reduction in LVEDD for each speed increase of − 0.14 cm/increment [6], as well as a narrowed gap between HVAD pump flow and CO (Table 1b and Fig. 1b). The patient was discharged uneventfully on his 165th day of hospitalization.

**Table 1:** (a) Result of echocardiographic and hemodynamic ramp test before AVR, (b) Result of echocardiographic and hemodynamic ramp test after AVR

HVAD monitor			TTE	RHC		
HVAD pump rate (rpm)	HVAD pump flow (L/min)	Power (Watts)	LVEDD (cm)	PCWP (mmHg)	CO (L/min)	CI (L/min/m2)
(a) Before AVR						
2300	3.7	3.0	5.86	24	3.05	1.86
2400	4.0	3.4	5.70	24	2.30	1.40
2600	4.4	4.2	5.52	21	2.30	1.40
2800	4.8	5.2	5.66	20	2.84	1.73
3000	5.1	6.3	5.73	18	3.00	1.83
3200	5.4	7.6	5.57	17	3.17	1.94
3400	5.7	9.1	5.71	15	3.48	2.13
(b) After AVR						
2300	3.0	2.6	5.60	18	2.88	1.78
2400	3.1	2.8	5.30	16	3.45	2.14
2600	3.7	3.6	5.20	13	4.05	2.51
2800	4.2	4.5	5.00	10	4.57	2.83
3000	4.5	5.5	4.90	7	6.03	3.74
3200	5.0	6.7	4.00	6	5.91	3.67
3400	5.5	8.1	4.00	8	7.51	4.66

A preoperative hemodynamic ramp test revealed only slight reduction in LVEDD for each speed and a correlation between increased HVAD pump rate and flow at each speed step. In contrast, there was a significant discrepancy between increased HVAD pump flow and CO at each speed step

Aortic valve was closed at the all times of test

*AVR* aortic valve replacement, *HVAD* HeartWare continuous-flow left ventricular assist device, *TEE* transthoracic echocardiography, *RHC* right heart catheter, *LVEDD* left ventricular end-diastolic diameter, *PCWP* pulmonary capillary wedge pressure, *CO* cardiac output, *CI* cardiac index

## Discussion

A severely regurgitant aortic lesion enables greater equalization of pressure between the aorta and left ventricle, compared with normal heart, thereby minimizing the pressure gradient and increasing the HVAD pump flow [7]. However, this increased HVAD pump flow does not match the increase in CO, because some amount of blood perfused by the HVAD returns to the left ventricle by a low-resistance circuit through the regurgitant aortic valve. In other words, we surmised that the difference of CO between the HVAD pump flow and RHC test arose from the blood flow returning to the left ventricle via a low-resistance circuit as a result of AI; the remaining blood enters the body and its flow is estimated by CO, calculated by RHC. Therefore, it might be useful to evaluate the discrepancy between HVAD pump flow and CO; it might give us a new perspective, and this could identify the causal relationship between AI and low CO in patients with CF-LVAD.

In the original echocardiography ramp test developed by Uriel et al. [8], AI was estimated by visual estimation of the severity in the parasternal long-axis view using the color Doppler imaging technique. For assessment of AI, the degree of regurgitation was graded from 0 to 6 (none to severe). However, this test might not allow simple estimation of the causal relationship between AI and low CO. Here, we performed a hemodynamic ramp test with simultaneous echocardiography and RHC. This hemodynamic ramp test revealed only slight reduction in LVEDD for each speed increase (− 0.00081 cm/increment) before AVR. This indicated that greater HVAD pump speed and flow did not contribute to the reduction of LVEDD. However, the contribution of AI to low CO remained unclear. Therefore, we performed simultaneous RHC to measure CO at each speed increase.

HVAD monitoring revealed a correlation between increased HVAD pump rate and flow at each speed step. In contrast, there was a significant discrepancy between increased HVAD pump flow and CO at each speed step; moreover, PCWP remained high (15 mmHg) at HVAD pump speed of 3400 rpm. We presumed that this discrepancy and high PCWP were related to the return of blood flow to the left ventricle through a low-resistance circuit, as a result of AI. Therefore, we decided that the patient was a suitable candidate for AVR. After AVR, his low CO symptoms disappeared; inotropes were successfully discontinued at 7 days after AVR. A postoperative hemodynamic ramp test revealed a significant reduction in LVEDD for each speed increase (− 0.14 cm/increment) and a smaller discrepancy between HVAD pump flow and CO at each speed step. PCWP decreased to 8 mmHg at HVAD pump speed of 3400 rpm. These findings indicated that elimination of AI by AVR successfully closed the loop of blood flow and reduced the discrepancy between HVAD pump flow and CO, thereby increasing CO.

In conclusion, a hemodynamic ramp test with simultaneous echocardiography and RHC was useful for the evaluation of the causal relationship between AI and low CO, and for the selection of surgical treatment for AI in a patient with CF-LVAD.
